# Peripheral Blood Mononuclear Cell Metabolism Acutely Adapted to Postprandial Transition and Mainly Reflected Metabolic Adipose Tissue Adaptations to a High-Fat Diet in Minipigs

**DOI:** 10.3390/nu10111816

**Published:** 2018-11-21

**Authors:** Yuchun Zeng, Jérémie David, Didier Rémond, Dominique Dardevet, Isabelle Savary-Auzeloux, Sergio Polakof

**Affiliations:** INRA, UNH, Unité de Nutrition Humaine, CRNH Auvergne, Université Clermont Auvergne, F-63000 Clermont-Ferrand, France; zengyuchunzlh@gmail.com (Y.Z.); jeremie.david@inra.fr (J.D.); didier.remond@inra.fr (D.R.); dominique.dardevet@inra.fr (D.D.); isabelle.savary-auzeloux@inra.fr (I.S.-A.)

**Keywords:** peripheral blood mononuclear cells, postprandial metabolism, high fat–high sugar diet, minipig, adipose tissue, biomarkers

## Abstract

Although peripheral blood mononuclear cells (PBMCs) are widely used as a valuable tool able to provide biomarkers of health and diseases, little is known about PBMC functional (biochemistry-based) metabolism, particularly following short-term nutritional challenges. In the present study, the metabolic capacity of minipig PBMCs to respond to nutritional challenges was explored at the biochemical and molecular levels. The changes observed in enzyme activities following a control test meal revealed that PBMC metabolism is highly reactive to the arrival of nutrients and hormones in the circulation. The consumption, for the first time, of a high fat–high sucrose (HFHS) meal delayed or sharply reduced most of the observed postprandial metabolic features. In a second experiment, minipigs were subjected to two-month HFHS feeding. The time-course follow-up of metabolic changes in PBMCs showed that most of the adaptations to the new diet took place during the first week. By comparing metabolic (biochemical and molecular) PMBC profiles to those of the liver, skeletal muscle, and adipose tissue, we concluded that although PBMCs conserved common features with all of them, their response to the HFHS diet was closely related to that of the adipose tissue. As a whole, our results show that PBMC metabolism, particularly during short-term (postprandial) challenges, could be used to evaluate the whole-body metabolic status of an individual. This could be particularly interesting for early diagnosis of metabolic disease installation, when fasting clinical analyses fail to diagnose the path towards the pathology.

## 1. Introduction

Peripheral blood mononuclear cells (PBMCs) have been widely used for more than 10 years now as a valuable tool to provide reliable biomarkers of health and diseases [1,2,3]. Although very recent studies have approached PBMC metabolism from a more functional point of view, such as metabolomics studies [4,5], most of them are based on transcriptomics analyses only [1,6,7]. Very little is actually known about the functional, i.e., biochemical-level, metabolism of PBMCs following nutrition-related interventions. Previous studies showed for specific individual white cell populations (such as lymphocytes and macrophages), these rapidly dividing cells had all the necessary biochemical machinery to utilise the most abundant energy substrates, such as glucose, lipids, and some amino acids, particularly glutamine [8]. More recent studies based on enzyme activities carried out on dogs, cats, and cattle confirm that PBMCs have an active metabolism that could be similar to that of other organs [9,10,11]. However, very little is known about PBMC metabolism in minipigs, as most of the studies are focused on immunological approaches. Only a few papers have explored the pig PBMC transcriptomic response to diverse conditions, such as mitogenic stimulation, [12], growth performance [13], stress [14], or leptin administration [15]. Thus, functional metabolic studies are lacking in minipigs, especially with respect to nutritionally-related metabolic diseases.

An aspect of interest in PBMCs is that they circulate permanently and through all body parts in the blood stream, being subjected to any variation of this fluid composition, including those related to fluctuations in circulating nutrients, substrates, and hormones [16]. All these molecules can then potentially impact PBMC metabolism and deeply modify their gene expression profile. On chronic exposure to a modified nutritional environment, PBMC transcriptomics have been used to discover valuable biomarkers of many metabolism-related diseases, such as diabetes [17], obesity [3], or insulin resistance [18]. Despite the current knowledge about the capacity of PBMCs to be imprinted at the long-term molecular level by altered nutritional conditions, it is not clear whether PBMC metabolism could be representative of nutritionally-induced short-term and rapid modifications in blood or tissue metabolites and metabolic activity. The postprandial phase (i.e., the period that follows meal intake) represents one of the most challenging phenomena in whole-body metabolism taking place in healthy conditions; it occurs every day, several times a day. Following meal intake, body metabolism must adapt to major changes in blood composition: the increased levels of circulating nutrients could be harmful remain elevated for a long time, and on the other hand, levels of several hormones increase for just few minutes after the nutrient arrival. As do all the other organs, PBMCs must adapt to these changes [19]. More importantly, while the contact of most of the organs with circulating nutrients and hormones will depend on vascularisation and changes in blood circulation, PBMCs are directly and permanently affected by the blood, as they are surrounded by the fluid. Changes in blood composition may be then particularly sharp for PBMCs, forcing them to rapidly adapt to these changes as suggested in transcriptomic-based postprandial studies [20,21]. For example, this has been demonstrated in studies on humans [22] and rats [16] during fasting, at least at the gene expression level. Whether this flexibility to adapt to a meal is compromised in PBMCs as has been shown for the whole body and individual organs during the onset of many metabolic diseases, such as diabetes or obesity [23,24], remains to be elucidated.

The main objective of the present study was to determine if PBMC metabolism at the biochemical and molecular levels could adapt to different nutritional conditions known to alter the profile of circulating metabolites and hormones, and to induce major changes in whole body metabolism. We submitted Yucatan minipigs to different nutritional challenges, including a meal test (a meal regular or high in fat and sugar, HFHS) and long-term HFHS feeding. In the first case, we aimed at determining whether PBMCs were able to adapt rapidly to changes occurring after meal intake and if their metabolic adaptive capacity might provide information about the capacity of the individual to handle the meal. In the second case we aimed at studying the long-term metabolic footprint of HFHS feeding on PBMC metabolism and to compare it to the metabolism of other tissues, including the liver, skeletal muscle, and adipose tissue (AT). For the postprandial challenges, the biochemical approach (enzyme activities) was chosen to explore the short-term adaptation (hours). For the long-term trial (months), both enzyme activities and mRNA levels were assessed to explore the metabolic adaptations installed in the fasting state during HFHS feeding.

## 2. Materials and Methods

### 2.1. Animals

The study involved 10 female adult (6 month-old) Yucatan mini-pigs (30 ± 1 kg). They were housed in subject pens (1 × 1.5 m) in a ventilated room with controlled temperature (21 °C) and regular light cycle (L12:D12). They were fed once daily with 400 g/day of a concentrate feed containing 17.5% proteins, 3.2% fat, 4.3% cellulose, and 5.2% ash (Porcyprima; Sanders Centre Auvergne, Aigueperse, France) and had free access to tap water. All procedures were in accordance with the guidelines formulated by the European Community for the use of experimental animals (L358-86/609/EEC, Council Directive, 1986).

### 2.2. Experimental Procedure

#### 2.2.1. Postprandial Meal Test

The postprandial meal test trial consisted in the ingestion of 400 g of a regular (control) or a high fat-high sugar (HFHS) meal after an overnight fasting period. Five animals were involved in the trial, consuming first the control diet, and after one week of wash-out, the HFHS diet. The HFHS diet consisted of a regular pig diet enriched with fat (12% butter) and sugar (10% sucrose). Animals ingested the whole mixture in no more than 10 min. Blood was collected though an arterial catheter before (T0) and then 2, 4, and 7 h after the meal.

#### 2.2.2. High Fat–High Sucrose Long-Term Trial

The HFHS trial consisted in the ingestion of an obesogenic diet during two months. Five animals were involved in the trial and were fed twice a day (500 g). Arterial blood was sampled through a permanent catheter after a fast of 24 h (day 0) and after 7, 14, 30, and 60 days of diet consumption also at the fasting state. Liver, skeletal muscle and AT samples were collected during the catheter surgery procedure (before the trial) and by the end of the 60 days. In both cases, minipigs were under deep anaesthesia and samples were immediately frozen at −80 °C for further analyses.

### 2.3. Analytical Procedures

#### 2.3.1. Blood Treatment and PBMC Collection

At each point, 10 mL of blood were collected on a dry syringe (without anticoagulant) and then gently transferred to a tube CPT™ tube (BD vacutainer) containing a polyester gel and a density gradient liquid (FICOLL™ Hypaque™ solution). This filter allows cell separation during a single centrifugation step. Briefly, blood was centrifuged at 1650 g during 20 min at 20 °C. After centrifugation, the plasma was collected and quickly stored at −80 °C until further analyses. The layer containing the PBMC was collected (buffy coat), washed twice with PBS solution and centrifuged at 300 g during 15 min at 20 °C. After washing, the PBMC were pelleted, the PBS discarded and cells suspended on RNLater™ (for further mRNA analysis) or a lysis solution.

#### 2.3.2. Plasma Insulin and Metabolite Determination

Glucose, triacylglycerol (TG), lactate, and urea levels were enzymatically measured using commercial kits on an automotive ABX Pentra 400 (Horiba Medical, Grabels, France) test system. Plasma insulin levels were assessed using a commercial ELISA kit (Mercodia, Uppsala, Sweden). Branched-chain amino acids (BCAA) were assessed enzymatically as described in Polakof et al. 2017 [25].

#### 2.3.3. Measurements of Enzyme Activities

For enzyme activities, PBMC were immediately homogenized on the lysis solution (20 mM Tris, pH 7.4, 250 mM sucrose, 2 mM EDTA, 10 mM β-mercaptoethanol, 100 mM NaF, 0.5 mM EDTA). The homogenate was centrifuged for 20 min at 10,000× *g* and the supernatant immediately used. Enzyme activities were determined by spectrophotometry using a microplate reader (Infinite^®^ 200 PRO NanoQuant, Tecan, Grödig, Austria), based on mini-pigs methods adapted to PBMC [23,25,26], including hexokinase (HK), pyruvate kinase (PK), glucose-6-phosphate dehydrogenase (G6PD), malate dehydrogenase (MDH), lactate dehydrogenase (LDH), glutamate dehydrogenase (GDH), glutaminase P-dependent, branched-chain amino acid aminotransferase (BCAT) and aspartate aminotransferase (AspAT).

#### 2.3.4. Western Blot Analyses

Samples were homogenized as described above and for each sample, total protein lysates (4 μg) were subjected to SDS-PAGE, electrotransferred on a PVDF membrane and probed with the indicated antibodies: total serine/threonine kinase protein kinase B (AKT) and phospho AKT at serine 473 (pAKT) and total eEF2-α and phosphor eEF2-α at threonine 56 (peEF2-α) (Cell Signaling Technology, Ozyme, St Quentin-en-Yvelines, France). After washing, membranes were incubated with an IRDye infrared secondary antibody (LI-COR Biotechnology, Lincoln, NE, USA). Bands were visualized by infrared fluorescence using the Odyssey imaging system (LI-COR Inc. Biotechnology, Lincoln, NE, USA) and quantified by Odyssey infrared imaging system software (version 1.2).

#### 2.3.5. PCR Analyses

Total RNA was extracted using RNEasy Mini Kit^®^ (Qiagen) and mRNA levels were determined by RT-PCR. RNA quality was verified on 1% agarose ethidium-bromide stained gel. cDNA was generated from 500 ng RNA using the High Capacity cDNA Reverse Transcription Kit (Life Technologies, Villebon-sur-Yvette, France). Real-time PCR was performed in the CFX96 Touch™ Real-Time PCR Detection System (BIO-RAD, Hercules, CA, USA) as in [23]. Primers were designed so that they are overlapping an intron (Primer3 software; Whitehead Institute for Biomedical Research/MIT Center, Cambridge, MA, USA) using known sequences in nucleotide databases. Primers sequences are available in [26].

### 2.4. Statistical Analyses

Data from the postprandial challenges were analysed using a repeated measures two-way ANOVA test (time and diet as variables). The PBMC and plasma parameters from the HFHS trial were analysed using a one-way ANOVA test followed by post-hoc Holm-Sidak. The *p*-value significance threshold for all factors was set to 0.05.

The differences between the D0 and D60 HFHS trial in liver, skeletal muscle and AT enzyme activities and mRNA levels were analysed using a Mann–Whitney nonparametric test using for the post hoc analysis the Student–Newman–Keuls test (SigmaPlot 12, Systat Software, San Jose, CA, USA). The *p*-value significance threshold for all factors and ions was set to 0.05. *p*-values between 0.05.

## 3. Results

### 3.1. Short-Term Control vs HFHS Postprandial Trial

**Figure 1:**
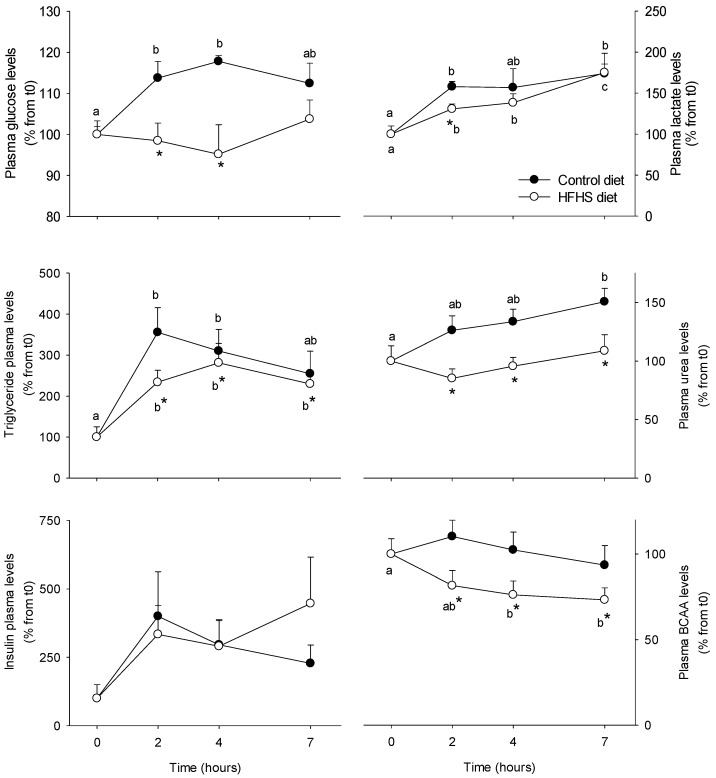
Plasma parameters in Yucatan mini-pigs fed either a control or a high fat–high sucrose (HFHS) test meals. Results are expressed as means + SEM (*n* = 5) and were analysed using a repeated measures 2-way ANOVA test. Different letters indicate significant differences between sampling points for a given meal test. * Significant different for a given sampling point between the control and the HFHS meals (*p* < 0.05). BCAA: branched-chain amino acids.

Glucose (insulin, glucose, lactate), lipid (TG), and nitrogen (urea, BCAA) metabolism were evaluated before and after the control and the HFHS meal based on plasma hormone and metabolite assessment in order to evaluate the postprandial metabolic phenotype of minipig followings the meal (Figure 1). Plasma glucose levels increased (between 2 and 4 h after the meal) after the control meal intake, recovering to basal level 7 h after the meal. No changes with time were observed following the HFHS meal, although plasma glucose levels were lower between 2 and 4 h than after the control meal. Plasma insulin levels showed a very important variability among individuals to put in evidence a time effect. However, 2 h after the meal, a tendency (*p* < 0.081) to have higher levels than before the meal was observed, which is in agreement with the changes observed in the plasma glucose profile. No differences were found among the animals consuming the different meals. Lactate plasma levels, a good indicator of glucose utilisation at the whole body level, increased significantly after both meal tests. However, the increase observed in the control group was more important, at least 2 h after the meal. Plasma TG levels increased also after the control and HFHS meals, but the maximum values were different among the groups. For the control group the peak was observed 2 h after the meal (+355%), while for the HFHS group it was delayed up to 4 h (+281%), most likely reflecting differences in the meal digestion and absorption. Postprandial urea plasma levels (indicative amino acid catabolism at the whole body level) showed different patterns: while in the control group levels steadily increased up to 151% at 4 h when compared to the fasting point, in the HFHS group no changes were observed. While BCAA levels after the control meal slightly increased (+12%), following the HFHS meal, their concentration was reduced up to 25% 7 h after the meal intake, suggesting their withdrawal from the blood compartment exceeded their appearance following the digestion process.

**Figure 2:**
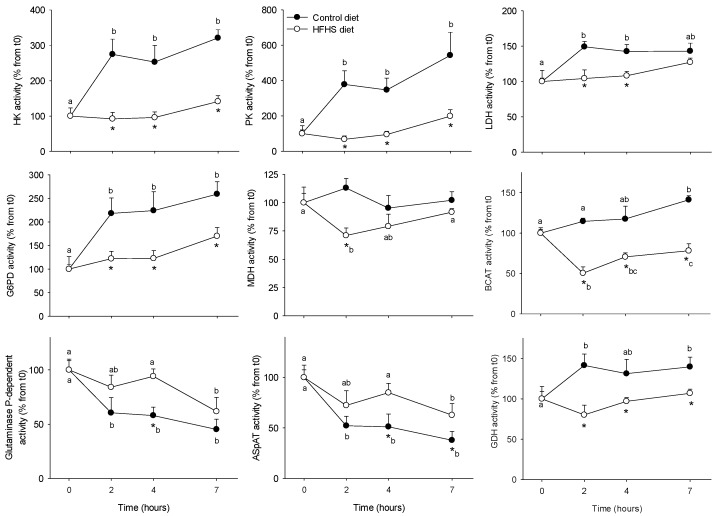
Hexokinase (HK), pyruvate kinase (PK), lactate dehydrogenase (LDH), glucose 6-phosphate dehydrogenase (G6PD), malate dehydrogenase (MDH), branched chain amino-acid transaminase (BCAT), glutaminase P-dependent, aspartate aminotransferase (AspAT) and glutamate dehydrogenase (GDH) alanine transaminase activities in PBMCs from Yucatan mini-pigs fed either a control or a high fat–high sucrose (HFHS) test meals. Enzyme activity units (mU) are defined as nmol of substrate converted to product, per min, at 37 °C and per mg protein. Results are expressed as means + SEM (*n* = 5) and were analysed using a repeated measures two-way ANOVA test. Different letters indicate significant differences between sampling points for a given meal test. * Significantly different for a given sampling point between the control and the HFHS meals (*p* < 0.05). PMBC: peripheral blood mononuclear cell.

**Figure 3:**
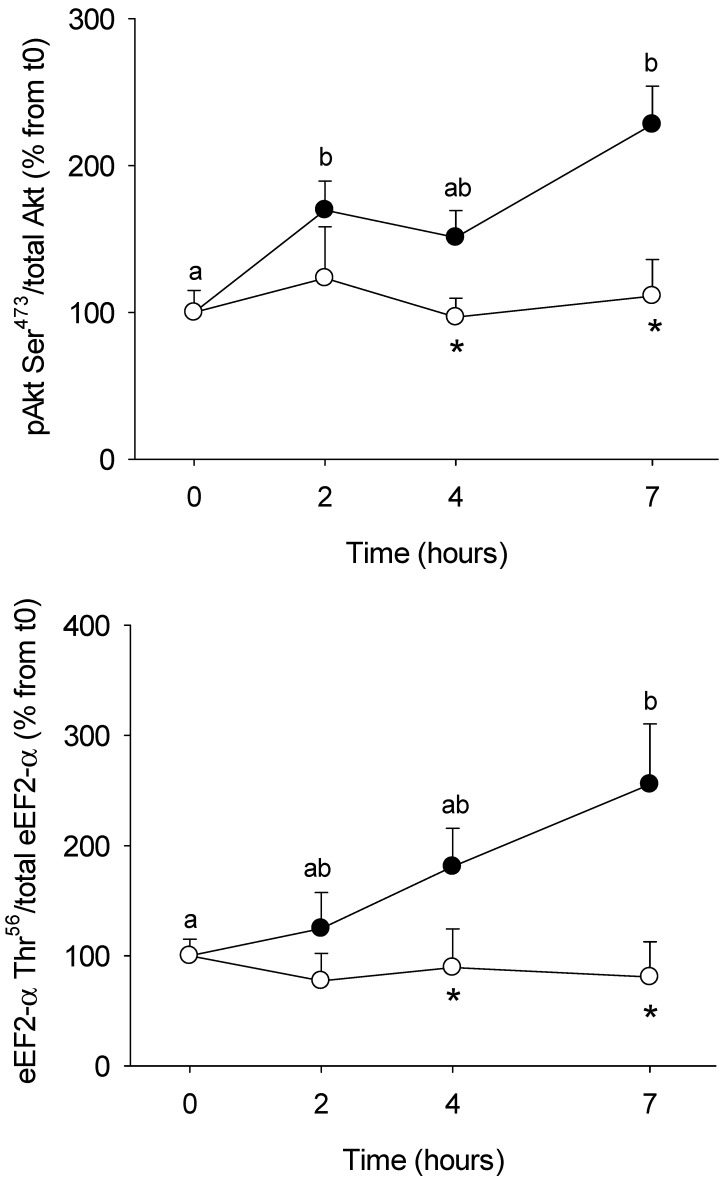
Phosphorylation levels of Akt Ser^473^ and eEF2-α Thr^56^ in PBMCs and S6 Ser^235/236^ from Yucatan mini-pigs fed either a control or a high fat–high sucrose (HFHS) test meals. Analysis was made by Western blot and levels of phosphorylated protein were normalized to the levels of the respective total protein (Akt Ser473 and eEF2-α). Results are expressed as means + SEM (*n* = 5) and were analysed using a repeated measures two-way ANOVA test. Different letters indicate significant differences between sampling points for a given meal test. *Significant different for a given sampling point between the control and the HFHS meals (*p* < 0.05).

Enzyme activities involved in glucose and amino acids metabolism in PBMC (Figure 2) were assessed in order to determine if they were affected by the meal (postprandial period) and its composition (regular vs. HFHS). We aimed also to explore if such changes could be related to the circulating metabolites and hormones described above. Two opposite profiles were observed: enzymes which activity increased after the control meal, including those participating in the glucose phosphorylation (HK, +300%), glycolysis (PK +400-600%, LDH, +150%), pentose phosphate pathway (G6PD, +200%), and amino acid catabolism (BCAT +125%, GDH, +140%); and enzymes for which activity was inhibited by the meal, such as those participating in amino acids metabolism, including glutaminase (−50%) and AspAT (−50%). For all of them, the response to the meal was further blunted when the HFHS test was performed. MDH activity (enzyme involved in the Krebs cycle) was not modified by the regular meal, but it was inhibited (−75% at 2 h) following the HFHS test, suggesting that those changes were meal-specific. Finally, we also explored the insulin (Akt) and protein synthesis (eEF2α) signalling pathways in order to evaluate the capacity of PBMC to transduce the nutritional stimuli from the meal into intracellular information able to regulate the glucose and protein metabolisms (Figure 3). In both cases, their phosphorylation statuses increased following the control meal (about +200% for both proteins), but no changes were observed after the HFHS test.

### 3.2. Long-Term HFHS Trial

After two months of HFHS feeding minipigs developed an obesity-like phenotype, with a significant increase in body weight (from 31.5 ± 1.4 kg to 44.7 ± 1.7 kg), most likely as the consequence of fat deposition at the visceral and subcutaneous adipose tissue [23]. The main metabolic features associated to this phenotype are described in Table 1. No changes were observed in glucose and lactate levels, while TG concentrations slightly increased between day 7 and 14. Insulin levels increased significantly from day 7 (about three times from day 0), and remained elevated up to the end of trial. In contrast, urea plasma levels were reduced from day 7 onwards (about 1.5 times) up to day 60. Finally, the inflammation status of minipigs was evaluated by assessing the CRP circulating levels at the beginning and at the end of the trial. CRP levels increased significantly from 0.52 ± 0.10 up to 0.99 ± 0.21 mg/L following the two months of HFHS feeding.

**Figure 4:**
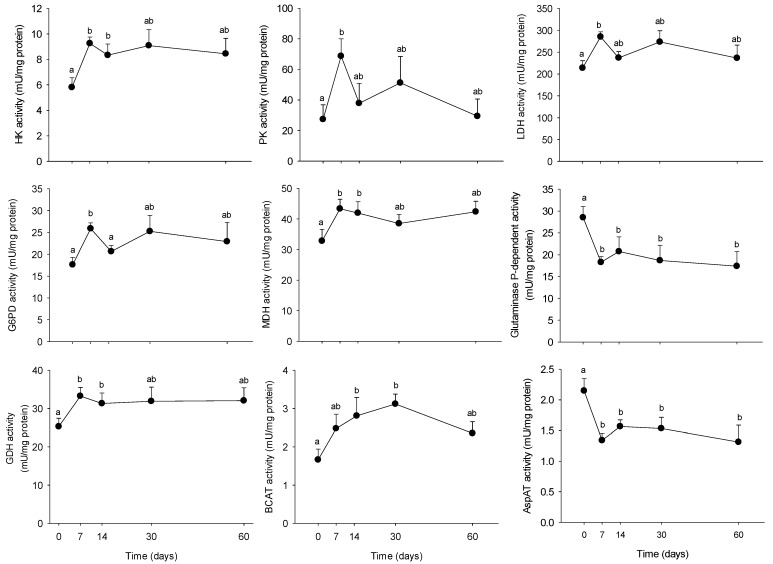
Hexokinase (HK), pyruvate kinase (PK), lactate dehydrogenase (LDH), glucose 6-phosphate dehydrogenase (G6PD), malate dehydrogenase (MDH), branched chain amino-acid transaminase (BCAT), glutaminase P-dependent, aspartate aminotransferase (AspAT), and glutamate dehydrogenase (GDH) alanine transaminase activities in PBMC from Yucatan mini-pigs fed a high fat-high sucrose (HFHS) diet for 2 months. Samples were obtained after an overnight fast period before (0) and 7, 14, 30, and 60 days after HFHS feeding. Enzyme activity units (mU) are defined as nmol of substrate converted to product, per min, at 37 °C and per mg protein. Results are expressed as means + SEM (*n* = 5) and were analysed using a one-way ANOVA test followed by post-hoc Holm-Sidak. Different letters indicate significant differences between the sampling points (*p* < 0.05).

**Figure 5:**
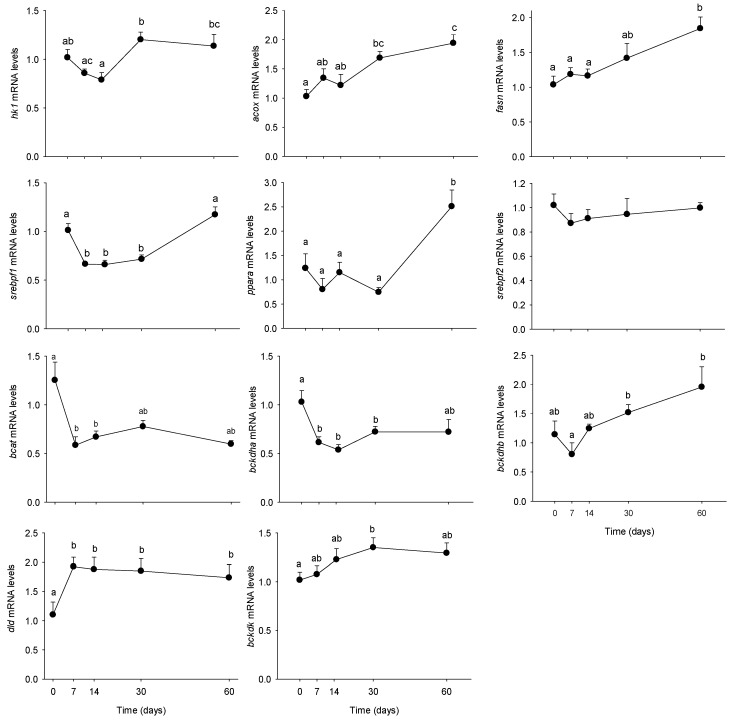
Hexokinase (*hk1*), acyl-coenzyme A oxidase (*acox*), fatty acid synthase (*fasn*), sterol regulatory element binding protein-1c (*srebf1c*), peroxisome proliferator-activated receptor alpha (*ppara*), sterol regulatory element binding protein-2 (*srebf2*), branched-chain alpha-keto acid dehydrogenase alpha/beta (*bckdha/b*), dihydrolipoyl dehydrogenase (*dld*), and branched chain ketoacid dehydrogenase kinase (*bckdk*) mRNA levels in PBMCs from Yucatan mini-pigs fed on high fat-high sucrose (HFHS) diet for 2 months. Samples were obtained after an overnight fast period before (0) and 7, 14, 30, and 60 days after HFHS feeding. Results are expressed as means + SEM (*n* = 5) and were analysed using a one-way ANOVA test followed by post-hoc Holm-Sidak. Different letters indicate significant differences between the sampling points (*p* < 0.05).

Once the metabolic phenotype of minipigs evaluated during the 2 months of HFHS feeding, we aimed at determining if the PBMC metabolism was able to respond to the modifications induced by the diet at the circulating level. We explored the PBMC metabolism at both, the enzyme activity and mRNA levels, as shown in Figure 4 and Figure 5, respectively. Overall, most of the explored enzymes increased their activities from day 7, recovering progressively their basal levels after 2 months of feeding. Only glutaminase and AspAT activities were reduced by the HFHS feeding (−50%), also from day 7. The tendency was conserved up to the end of the trial. Concerning the molecular analyses, several genes resulted up-regulated by the long-term HFHS feeding, including *acox, fasn, bckdhb* and *bckdk*, for which levels increased progressively with time. Only *dld* mRNA levels increased at day 7 and remained stable up to the end of the trial. *Bcat* and *bckdhb* mRNA levels were down-regulated by the HFHS consumption, while no changes were observed on *srebpf2* levels. Finally, *srebpf1* levels were transiently reduced between day 7 and 30 (−40%) but recovered the initial levels at day 60.

**Figure 6:**
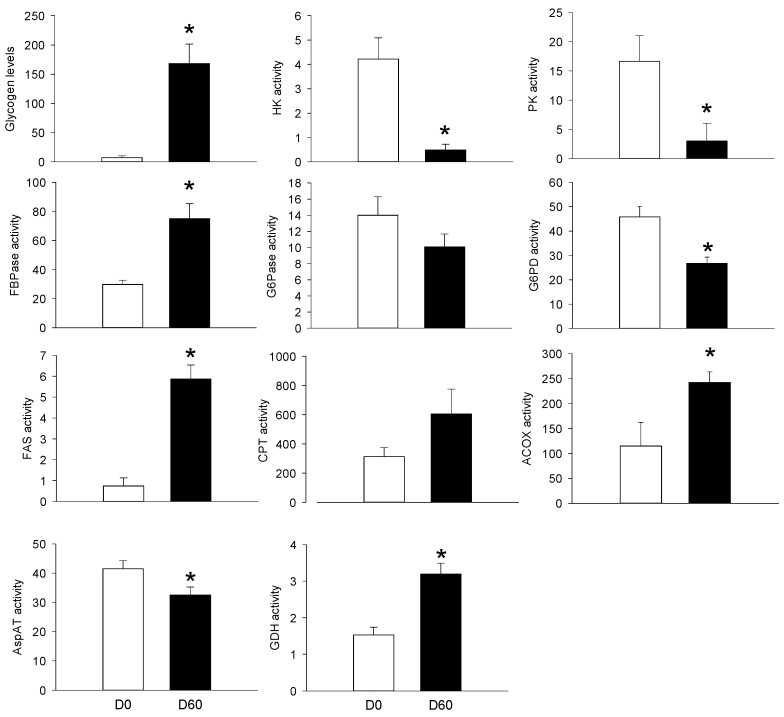
Glycogen levels, hexokinase (HK), pyruvate kinase (PK), fructose 1,6-biphosphatase (FBPase), glucose 6-phosphatase (G6Pase), glucose 6-phosphate dehydrogenase (G6PD), fatty acid synthase (FAS), carnitine palmitoyltransferase (CPT), acyl-CoA oxoxidase (ACOX), aspartate transaminase (AspAT), and glutamate dehydrogenase (GDH) activities in liver samples from Yucatan mini-pigs fed a high fat–high sucrose (HFHS) diet for 2 months. Enzyme activity units (mU) are defined as nmol of substrate converted to product, per min, at 37 °C and per mg protein. Glycogen levels are expressed in μmol of glycosyl units/g wet tissue. Results are expressed as means + SEM (*n* = 5) and were analysed using a t-student test. *, significantly different from the D0 (*p* < 0.05).

**Figure 7:**
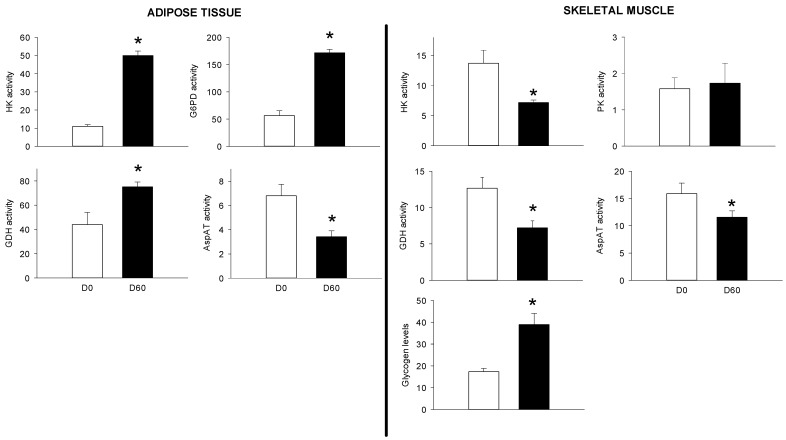
Glycogen levels, hexokinase (HK), pyruvate kinase (PK), glucose 6-phosphate dehydrogenase (G6PD), aspartate transaminase (AspAT), glutamate dehydrogenase (GDH) activities in adipose tissue and skeletal muscle samples from Yucatan mini-pigs fed a high fat–high sucrose (HFHS) diet for 2 months. Enzyme activity units (mU) are defined as nmol of substrate converted to product, per min, at 37 °C and per mg protein. Glycogen levels are expressed in μmol of glycosyl units/g wet tissue. Results are expressed as means + SEM (*n* = 5) and were analysed using a *t*-student test. *, significantly different from the D0 (*p* < 0.05).

The third objective of the present study was to compare PBMC metabolic potential to those of other organs, such as the liver, the skeletal muscle and the AT. We therefore explored the metabolism of those tissues before and 2 months after HFHS feeding. As for the PBMCs, we choose a double approach, including biochemical (glycogen levels, enzyme activities) and molecular (mRNA levels) targets as presented in Figure 6 and Figure 7, as well as in Table 2, respectively. At the hepatic level, the HFHS feeding resulted in increased glycogen levels (+24-fold), and in enzymes participating at the gluconeogenesis (FBPase, +3-fold), lipogenesis (FAS, +5-fold), lipid oxidation (Acox, +2.5-fold) and amino acids catabolism (GDH, +3-fold). Other enzyme activities were inhibited by the HFHS feeding, including those participating at the glycolysis (HK, PK, about −4-fold), the pentose phosphate pathway (G6PD, −1.8-fod), and amino acid transamination (AspAT, −1.3-fod). Except for the AspAT activity (which was inhibited (−2-fold)), all the explored enzymes were up-regulated in the adipose tissue, including HK (+5-fold), G6PD (+3-fold) and GDH (+2-fold). At the muscle level, glycogen levels increased by 2-fold after 2 months of HFHS feeding, while HK, GDH, and AspAT activities were reduced.

Concerning the mRNA levels of proteins involved in glucose, lipids and amino acids metabolism are at the hepatic level, the general tendency was the reduction of expression induced by the HFHS feeding including those genes involved in glucose metabolism such as *hk1* (−1.5-fold) and *g6pc* (−2-fold), or in lipid oxidation like *cptl* (−5-fold) or lipogenesis (*acly*; −1.3-fod). The only exception was another gene involved in lipogenesis, *fasn*, which mRNA levels were dramatically (+23-fold) increased by the HFHS diet intake. Only minor changes were observed in the skeletal muscle, with a 50% reduction it the mRNA levels of the *cptm*, involved in β-oxidation. Several genes were up-regulated by the HFHS feeding in the AT, including those participating at the glucose transport, like *slc2a4* (+6-fold), *hk1* (+1.4-fold), or lipogenesis, like *acly* (+10-fold) and *fasn* (+9-fold). In contrast, the hepatic isoform of CPT (*cptl*) was down-regulated (−5-fold) the HFHS feeding.

## 4. Discussion

In the present study we show for the first time that the metabolism of mini-pig PBMC can be altered at the biochemical and molecular levels and that it was closely related to the AT profile, at least in the context of a long-term HFHS feeding. Further, we showed that the PBMC metabolism, particularly at the biochemical level, can be modulated at the postprandial level.

### 4.1. PBMC Metabolism Is Highly Reactive to the Meal, But Its Response Is Blunted by the Intake of a HFHS Meal

After meal intake, the body must adapt to the greater influx of nutrients in a very short period of time (from minutes to a couple of hours). Although several studies have previously explored the postprandial response of PBMC, they were focussed on transcriptomics analyses only [20,21,27]. Given the very short-term response needed after the meal, we choose to explore the postprandial metabolism at the biochemical and signalling levels rather than at the molecular level, which as far as we are aware has never been done before. The objective was therefore to elucidate if PBMC metabolism could respond in the very short-term to a nutritional stimulus and provide valuable information about changes at the whole body metabolism and metabolic flexibility that could not be visible at the fasting state [16,28]. In our study, PBMC metabolism was indeed highly responsive to the control meal challenge. The glucose phosphorylation potential and further channelling through the glycolysis was strongly increased, highlighting the importance of glucose for fuelling PBMC metabolism [29]. This was further supported by the meal-stimulated phosphorylation of Akt, a major actor on the insulin signalling pathway. Other metabolic processes were also stimulated by the meal, as the NADPH production to support lipogenesis or nucleotide biosynthesis through the pentose phosphate pathway.

PBMCs not only rely on glucose as energy source, as glutamine utilization in these cells can be even greater than that of glucose [8]. In our study glutaminase activity was significantly reduced following the meal, in line with its increased activity in lymphocytes from starved rats [29]. The fact that the AspAT activity followed the same profile than the glutaminase, further confirms that this enzyme rather than GDH is mainly responsible of the glutamate to α-ketoglutarate conversion in PBMCs. However, PBMCs do not only use glutamine for energy purposes, as the production of non-essential amino acids (such as alanine and aspartate) via the transaminases for biosynthetic purposes is of great importance for this kind of highly proliferating cell [8]. Following the meal, when circulating amino acid levels increase, PBMCs would rather rely on them for biosynthesis processes, reducing the demand from transaminases and resulting in an increase in the glutamate channelling through the GDH reaction [30], explaining the increased GDH activity in our study. Finally, the fact that the glucose utilisation was enhanced in response to the meal, whereas glutamine utilisation was reduced, strongly suggests that during the fasted-to-fed transition a metabolic shift allows PBMC to switch from glutamine to glucose utilisation [31].

During the HFHS meal test, we surprisingly found that the PBMC response was often blunted or in some cases delayed when compared to the regular test meal. The postprandial trial aimed at challenging the metabolic response of PBMC, which has been achieved with the control meal. By providing for the first time an HFHS meal to the same animals, our goal was to further alter the PBMC environment in terms of composition and time-course appearance of nutrients and hormones. Thus, the kinetics of most of the plasma parameters were altered following the HFHS meal, most likely due to the impact of the diet on gut physiology (motility, gastric emptying) and hormone release (CCK, PYY, GLP-1) [32]. Thus, despite a higher content of sugar than in the regular meal, the HFHS consumption did not result in a significant postprandial increase in blood glucose levels. Accordingly, the same lack of responsiveness was observed in the insulin signalling pathway, resulting (together with the low glucose available) in a non-stimulated glucose utilisation, and supporting the idea that PBMC strongly depend on circulating substrates, particularly glucose. This could explained in part by a delayed and reduced excursion of glucose and insulin mediated by a slower gastric emptying and reduced gut hormone secretion, as previously observed in humans subjected to high fat diet tests [33,34,35].

Unlike after the control meal, following the HFHS intake we did not longer observe the fasting-to-fed transition between glucose and glutamine utilisation. This suggests that PBMC metabolism has adapted to the reduced glucose levels and continue to rely on glutamine, such as during the post-absorptive period. In contrast to other circulating metabolites, BCAA levels following the HFHS meal showed reduced levels during the postprandial period, which resulted in a lower transamination potential as well, likely reducing the flux of ketoacids up to the Krebs cycle. In agreement with this, the phosphorylation status of the protein eEF2α, responsible for determine the end of the translation and sensitive to the leucine signal role, remained unchanged after the HFHS meal. This may represent another sign of the altered response to the meal stimulus. Another symptom of impaired normal metabolic activity after the HFHS meal was the reduction on MDH activity. Since MDH plays crucial roles in the malate–aspartate shuttle, essential for coupling activation of mitochondrial and energy production [36], a decrease in MDH activities may suggest a depression of the transfer of NADH, and less ATP production in mitochondria.

### 4.2. Time-Course Changes in PBMC Metabolism during Long-Term High Fat Feeding

The second goal of our study was to evaluate if the metabolism of PBMC could be modified by a long-term nutritional change, such as HFHS feeding-induced obesity. As in our previous study focused on tissue/organ metabolism [23], we observed in the present study that the same major metabolic changes occurred also in PBMC after only one week of HFHS feeding. However, unlike during the short-term stimulation (postprandial period) PBMC do not seem to rely on available circulating metabolites during the long-term HFHS imprinting. Thus, despite the lack of changes in post-absorptive blood glucose levels, PBMC glucose metabolism was enhanced, as previously shown in a PBMC transcriptome-based study comparing lean vs. obese subjects [20]. It is known that alterations in postprandial glucose kinetic precedes those at the fasting state in the insulin resistance installation [37]. The increased glucose metabolism at the fasting state observed in the circulating PBMC may be the result of a long-term important glucose postprandial excursions (as supported by the hyperinsulinemia), exposing the circulating cells to elevated postprandial glucose levels. If our results are further investigated in future studies, alterations in these enzyme activities could constitute early events signalling the beginning of glucose alterations leading to insulin resistance, even when fasting blood glucose levels remain unchanged.

PBMCs seem to also have very active lipid metabolism [22]. Thus, genes involved in lipid catabolism, such as *acox* and *ppara*, were up-regulated by the long-term HFHS feeding, suggesting that minipig PBMC metabolism adapted to the increased dietary lipids, as previously observed in PBMCs from obese rats fed on cafeteria diets [2,38]. On the other hand, markers of lipogenesis (*fasn, srebpf1*) also resulted up-regulated. A recent study showed that lipogenesis was active in PBMCs and that was highly dependent on glucose [39], a strong activator of lipogenic genes [40]. This effect of glucose on de novo lipid synthesis represents a common feature for lipogenic organs, such as the liver or the AT from HFHS-fed animals [28,41,42].

As far as we are aware, the metabolism of amino acids has never been explored on PBMC from obese animals. After two months of HFHS feeding, both glutaminase and AspAT activities resulted rapidly (one week) down-regulated, suggesting that amino acids use for energy was reduced in detriment of other alternative fuels like glucose and lipids [23]. BCAA related-enzyme activities and mRNA levels were also reduced by the end of the trial, supporting the idea of a progressively reduction of BCAA catabolism from obese minipigs. We and other have shown that in obese and insulin resistant animal models, BCAA circulating levels increased with time [25,43]. Interestingly, the increased transamination potential during the first weeks of HFHS feeding could be the consequence of a metabolic adaptation to the progressively increase in BCAA levels. However, by the end of the trial, the potential to catabolise BCAA was blunted, in agreement with a defective oxidative deamination observed in the adipose tissue of HFHS-fed minipigs [25].

### 4.3. The PBMC Metabolism Rather Resembled that of the Adipose Tissue

As explained above, PBMC metabolism is different from any other organ, as these cells are actually permanently stimulated by changes in circulating blood metabolites and hormones. Despite this, we demonstrated that their metabolism has many common features with several major organs of the body. The second objective of the present study was therefore to compare PBMC response to the long-term HFHS meal to that of the liver, skeletal muscle and the AT.

We observed that an adaptive strategy was settled at the hepatic level in order to handle the excess of nutrients and energy brought by the HFHS diet. Thus, those pathways involved in glucose utilisation, such as glycolysis and pentose phosphate pathway were reduced, while lipid oxidation potential increased, illustrating a metabolic switch aiming at giving the priority to the more abundant energy substrates available. In accordance with this, we observed that the liver increased its capacity to store the excess of energy in the form of lipids (lipogenesis) and glycogen. Of note, the increased FBPase activity and reduced G6Pase potential strongly suggests that the glucose produced via the gluconeogenesis was further stored as glycogen rather than being exported into the blood stream. Unlike the liver, the overall metabolic features observed in the skeletal muscle and the AT suggest that both organs enhanced the uptake, utilisation, and storage of glucose. Two other catabolic features were also different from those of the liver, including lipids and amino acids oxidation pathways, that were reduced in the muscle and the AT. As for the liver, lipogenesis potential was strongly up-regulated in the AT. The integrative view of the HFHS minipig physiology suggests therefore that at the whole-body level, inflammation was settled. Despite this, the metabolism seemed flexible enough to adapt and kept blood glucose levels into within the physiological range. This was achieved through a coordinated increased in glucose uptake, utilisation and storage from all the explored organs in order to remove as much as possible glucose from the blood circulation. Further, the liver and the AT increased also their potential of other highly consuming glucose pathways, such as *de novo* lipogenesis. Finally, the lipid handling seemed to be organ-dependent, as the liver increased the oxidation potential of lipids, while the muscle and the adipose tissue showed an opposite trend.

When compared to the results discussed above, the metabolic response observed in the PBMCs in the obese minipig at the fasting state seems to be coherent with the adaptations reported in the major organs involved in the intermediary metabolism. As in a previous study in hamsters [44] our results show that PBMC metabolism does not reflect that of the liver. In contrast, the metabolic profile of PBMC evaluated at the molecular and biochemical levels was particularly close to the one observed in the AT. This is in line with similar conclusions draws in several studies in humans [45] and laboratory animals [16,44], specially concerning lipid metabolism [46]. In contrast to this, other metabolic features, like pentose phosphate potential seems to have a PBMC-specific behaviour, with a strong dependence on glucose uptake and phosphorylation and a lower induction capacity when compared to the AT. The responsiveness of PBMCs, particularly at the biochemical level could be then potentially used to explore the whole body metabolism in response to different dietary interventions or to evaluate the functional metabolic status of an individual with a single blood sample.

## 5. Conclusions

As a whole, the evaluation of the metabolic response to a control test meal of PBMC showed for the first time at the biochemical level that these cells were able to modify and adapt in a very short period (often < 3 h) their metabolic activity under the influence of circulating nutrient and hormones. Most of the changes recorded showed that PBMCs relayed glutamine metabolism during the post-absorptive period, and that they switched to glucose when quantity of carbohydrates from the diet increased in the circulation. Interestingly, we showed that major and fast changes in the PBMC environment induced by the HFHS during the postprandial period were able to trigger in PBMCs the necessary metabolic changes to adapt to this challenge. Finally, the exploration of the PBMC metabolism in long-term HFHS feeding confirmed that the regulation pattern observed in PBMCs fits with that expected in tissues involved in energy balance. It would be therefore reasonable to use them to evaluate whole-body metabolism without the need to perform invasive organs biopsies, as they can act as metabolic sentinels of nutritional-related changes. The common features observed between the PBMC and AT metabolism in HFHS minipigs make these cells particularly attractive for exploring metabolism and looking for biomarkers of dysregulation in obesity-related conditions.

## Figures and Tables

**Table 1:** Plasma fasting metabolites in Yucatan minipigs fed a high fat–high sucrose diet (HFHS) over 2 months.

	*Time (Days)*				
	*1*	*7*	*14*	*30*	*60*
*Glucose* (mM)	3.78 ± 0.28	3.54 ± 0.11	3.42 ± 0.12	3.56 ± 0.15	3.54 ± 0.07
*Lactate* (mM)	0.53 ± 0.07	0.51 ± 0.03	0.49 ± 0.03	0.48 ± 0.04	0.58 ± 0.06
*Triglycerides* (mM)	0.19 ± 0.03a	0.35 ± 0.06b	0.33 ± 0.07b	0.21 ± 0.03a	0.32 ± 0.05ab
*Insulin* (ng/mL)	0.05 ± 0.03a	0.17 ± 0.04b	0.16 ± 0.04b	0.17 ± 0.05b	0.14 ± 0.03b
*Urea* (mM)	5.38 ± 0.43a	3.41 ± 0.32b	3.79 ± 0.15b	3.92 ± 0.31b	4.06 ± 0.31b

Results are expressed as means + SEM (*n* = 5) and were analysed using a one-way ANOVA test followed by post-hoc Holm-Sidak. Different letters indicate significant differences between the sampling points (*p* < 0.05).

**Table 2:** mRNA levels of proteins involved in glucose and lipid metabolism in the liver, adipose tissue and skeletal muscle samples from Yucatan minipigs fed on a high fat–high sucrose diet (HFHS) over 2 months.

	*Liver*			*Skeletal Muscle*			*Adipose Tissue*		
*Metabolic Pathway*	*Gene*	*D0*	*D60*	*Gene*	*D0*	*D60*	*Gene*	*D0*	*D60*
**Glucose metabolism**	*gck*	0.63 ± 0.20	1.57 ± 0.54	*slc2a4*	1.03 ± 0.12	1.42 ± 0.14	*slc2a4*	1.03 ± 0.11	6.40 ± 1.69 *
	*g6pc*	1.51 ± 0.21	0.78 ± 0.15 *						
	*pck1*	1.09 ± 0.09	0.94 ± 0.07	*hk-1*	0.70 ± 0.08	1.50 ± 0.37	*hk-1*	1.01 ± 0.06	1.45 ± 0.16 *
	*hk1*	1.23 ± 0.10	0.84 ± 0.05 *						
**Lipid oxidation**	*cpt1-l*	2.43 ± 0.30	0.44 ± 0.04 *	*cpt1-l*	1.03 ± 0.11	0.50 ± 0.06 *	*cpt1-l*	1.27 ± 0.26	0.27 ± 0.04 *
	*acox*	1.01 ± 0.06	0.86 ± 0.05	*acox*	0.87 ± 0.04	0.82 ± 0.09	*acox*	1.03 ± 0.11	0.79 ± 0.17
	*ppara*	1.25 ± 0.34	1.03 ± 0.09	*ppara*	1.02 ± 0.09	0.73 ± 0.08 *^t^*	*ppara*	1.04 ± 0.14	1.46 ± 0.18 *^t^*
**Lipogenesis**	*acly*	1.01 ± 0.08	0.75 ± 0.04 *	-			*acly*	1.16 ± 0.34	11.88 ± 1.29 *
	*fasn*	0.23 ± 0.03	5.37 ± 1.19 *				*fasn*	1.19 ± 0.36	10.34 ± 2.57 *
	*srebf1c*	0.92 ± 0.16	1.27 ± 0.21				*srebf1c*	1.29 ± 0.47	0.61 ± 0.05

Each value is the mean ± SEM of *n* = 5 animals. mRNA levels were estimated using real-time RT-PCR. mRNA expression values were normalized with β-actin expressed transcripts and are indicated as fold variation of the control group. * significantly different from D0 (*p* < 0.05) among groups. *gck*, glucokinase; *pck1*, phosphoenolpyruvate kinase; *g6pc*, glucose 6-phosphatase; *acly*, ATP-citrate lyase; *fasn*, fatty acid synthase; *srebf1c*, sterol regulatory element binding protein-1c; *cpt1-l/m*, carnitine palmitoyltransferase-liver/muscle isoform; *acox*, acyl-coenzyme A oxidase; *ppara*, peroxisome proliferator-activated receptor alpha; *hk-1*, hexokinase 1.
